# Application of a neural network model in estimation of frictional features of tribofilms derived from multiple lubricant additives

**DOI:** 10.1038/s41598-024-62329-z

**Published:** 2024-05-22

**Authors:** Hiroshi Noma, Saiko Aoki, Kenji Kobayashi

**Affiliations:** 1 Department of Chemical Science and Engineering, School of Materials and Chemical Technology, Tokyo Institute of Technology, S1-31, 12-1 O-Okayama 2-Chome, Meguro-ku, Tokyo, 152-8552 Japan; 2 Idemitsu Kosan Company, Ltd., 24-4 Anesakikaigan, Ichihara-shi, Chiba 299-0107 Japan

**Keywords:** Cheminformatics, Mechanical engineering

## Abstract

In the field of tribology, many studies now use machine learning (ML). However, ML models have not yet been used to evaluate the relationship between the friction coefficient and the elemental distribution of a tribofilm formed from multiple lubricant additives. This study proposed the possibility of using ML to evaluate that relationship. Friction tests revealed that, calcium tribofilms formed on the friction surface, with the friction coefficient increasing as a result of the addition of OBCS. Therefore, we investigated whether the convolutional neural network (CNN) model could recognize the tribofilms formed from OBCS and classify image data of the elemental distributions of these tribofilms into high and low friction-coefficient groups. The CNN model classifies only output values, and it’s difficult to see how the model has learned. Gradient-weighted class activation mapping (Grad-CAM) was performed using a CNN-based model, and this allowed the visualization of the areas important for classifying elemental distributions into friction coefficient groups. Furthermore, dimension reductions enabled the visualization of these distributions for classification into the groups. The results of this study suggested that the CNN model, the Grad-CAM, and the dimension reductions are useful for evaluating frictional features of tribofilms formed from multiple lubricant additives.

## Introduction

New methods to reduce carbon dioxide (CO_2_) emissions, the principal driver of global warming, are urgently needed. One critical approach is to reduce friction loss in machinery, a major source of CO_2_ emissions^1^. This is generally accomplished by tailoring the profiles of lubricant additives for a specific machine^2^.

Continuously variable transmissions (CVTs) have been widely implemented in automobiles to improve their fuel efficiency. CVTs transfer power from the car engine to the wheels via the friction in the metal-to-metal contacts between the CVT belt and pulleys. To further enhance CVT transmission efficiency, it is thus necessary to increase friction at the metal-to-metal contact points between the belt and pulleys. To that end, lubricating oils known as CVT fluid (CVTF) play an important role. CVTFs usually consist of a base oil and various kinds of lubricant additives^3,4^. Among these additives, phosphorus- and sulfur-based extreme pressure (EP) additives and calcium-based detergents influence the friction properties at the metal-to-metal contacts between the CVT belt and pulleys. By appropriately combining phosphorus-, sulfur-, and calcium-based additives, tribofilm formation can be controlled and a high friction coefficient can be achieved^5–10^. However, it remains to be elucidated how tribofilm that is formed from CVTF, which contains such additives, provides high friction.

Many studies have investigated the chemical properties of tribofilms formed from lubricant additives to elucidate the friction mechanism. Some studies have utilized X-ray photoelectron spectroscopy (XPS)^11–13^ and time-of-flight secondary ion mass spectrometry (ToF-SIMS)^14^. In the present study, we conducted friction tests with a laboratory-made high-frequency reciprocating tribometer (HFRT) to measure the coefficients of friction on a tribofilm formed from sample oils formulated with combinations of tricresyl phosphate (TCP), dibenzyl disulfide (DBDS), and overbased calcium sulfonate (OBCS) as phosphorus-, sulfur-, and calcium-based additives, respectively. After the friction tests, the elemental distributions of the tribofilms were analyzed by electron probe micro-analysis (EPMA). The friction test results showed that, the calcium tribofilms formed on the friction surface and that the addition of OBCS increased the friction coefficient. This suggests that the formation of thick tribofilms is related to high friction through the addition of OBCS^15^. However, it was suggested that the relationships between the chemical properties of the tribofilm and the friction coefficient are not one-to-one but rather intricately intertwined. Therefore, we considered that machine learning (ML) could be used to investigate the friction mechanism in a comprehensive manner.

ML has been applied across various academic fields and has contributed significantly to many studies. Within the field of tribology, ML models have been constructed to predict fault diagnosis^16–21^, estimate life^22,23^, determine lubrication regimes^24^, and analyze wear properties^25^ from sensor datasets. In addition, ML models have been developed to predict wear properties^26–32^, friction coefficients^26,31^, and surface morphologies^29,33^ from laboratory-scale experimental datasets. Moreover, ML models have been built to develop the lubricating oils^34,35^. The results of these studies suggest that ML models were able to express various relationships between input and output values. Thus, tribological application of ML-based models has been extensive, and their use in this field is expected to continue to expand^36^.

The present study used a convolutional neural network (CNN) because the image data of the elemental distributions of tribofilms were used as the input values. The CNN, which is a kind of neural network, is used to predict the output value from image data. This is because neural network models predict only output values from per-pixel values of image data, whereas CNN models can recognize features from some parts of the image data. Many studies in tribology have used CNN models^37–39^, but CNN models have not been constructed to investigate the relationship between the elemental distributions of tribofilms and their friction coefficients. The disadvantage of CNN models is that it is impossible to see how they learn. Therefore, a gradient-weighted class activation map (Grad-CAM) was used in this study^40^. A Grad-CAM can visualize the areas in the image data that are important for predicting output values. Grad-CAMs have been used in many studies^41,42^, though rarely in the field of tribology. Furthermore, dimension reductions were used to classify the image data of the elemental distributions of tribofilms in this study. Dimension reductions have been used not only to increase ML learning speed^17,20^ but also to visualize the distribution of high-dimensional data^43,44^. However, dimension reductions have not yet visualized the distribution of tribofilm image data to investigate the relationship between the elemental distribution of a tribofilm and its friction coefficient.

Therefore, the purpose of this study is to investigate the possibility of using the CNN model, Grad-CAM, and dimension reductions to evaluate the relationship between the friction coefficient and the elemental distribution of a tribofilm that contains multiple lubricant additives. The friction tests revealed that adding OBCS led to the formation of calcium tribofilms and increased their friction coefficients. Therefore, we investigated whether the CNN model and dimension reductions were able to classify the image data of the elemental distributions of tribofilms into high and low friction-coefficient groups. We also used Grad-CAM to investigate whether the CNN model could recognize the tribofilms that formed from OBCS in order to perform the classification.

## Methods

### Study flow

**Figure 1:**
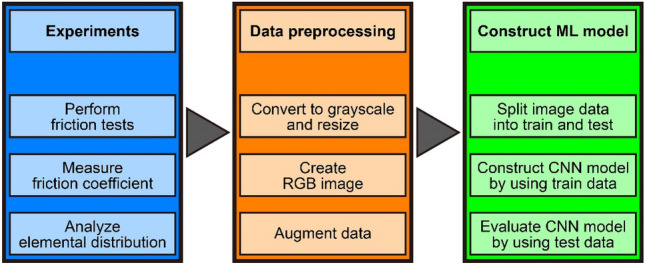
Framework of the research methodology used in this study.

The framework of the research methodology used in this study is diagrammed in Fig. 1. A CNN model was constructed to classify the image data of the elemental distributions of tribofilms into high and low friction-coefficient groups. The friction coefficient was measured by friction tests, and the elemental distributions of tribofilms were analyzed by chemical analysis. RGB image data were created from the elemental distributions, and data were augmented to increase the amount of image data. Furthermore, the image dataset was split into a train dataset and a test dataset. The train dataset was used to construct the CNN model, while the test dataset was used to evaluate the CNN model. The following sections explain the experiments, the data preprocessing, and the construction of the CNN model in detail.

### Sample oil

Polyalphaolefin (PAO), tricresyl phosphate (TCP), dibenzyl disulfide (DBDS), and overbased calcium sulfonate (OBCS) were used as an additive-free base oil and as phosphorus-, sulfur-, and calcium-based additives, respectively. PAO had a kinematic viscosity of 3.88 mm^2^/s at 100 °C. TCP was appended to the base oil at phosphorus concentrations of 0.025, 0.05, and 0.10 mass%, and then unary solutions of TCP were prepared for the friction tests. Furthermore, DBDS and OBCS were appended to a solution including only TCP at elemental concentrations of 0.025, 0.05, and 0.1 mass%, and then binary and trinary solutions of TCP and the other additives were prepared for the friction tests. Table 1 shows the concentrations of the lubricant additives in each sample oil. Thirty-six sample oils were prepared in this study.

**Table 1:** Below is a list of sample oils used in the friction tests. The friction test results for each sample oil were used to construct the machine learning models.

Sample oil	TCP, mass%	DBDS, mass%	OBCS, mass%
TCP0.025	0.025		
TCP0.05	0.05		
TCP0.1	0.1		
TCP0.025 + OBCS0.025	0.025		0.025
TCP0.025 + OBCS0.05	0.025		0.05
TCP0.025 + OBCS0.1	0.025		0.1
TCP0.05 + OBCS0.025	0.05		0.025
TCP0.05 + OBCS0.05	0.05		0.05
TCP0.05 + OBCS0.1	0.05		0.1
TCP0.1 + OBCS0.025	0.1		0.025
TCP0.1 + OBCS0.05	0.1		0.05
TCP0.1 + OBCS0.1	0.1		0.1
TCP0.025 + DBDS0.05	0.025	0.05	
TCP0.05 + DBDS0.025	0.05	0.025	
TCP0.05 + DBDS0.05	0.05	0.05	
TCP0.05 + DBDS0.1	0.05	0.1	
TCP0.1 + DBDS0.025	0.1	0.025	
TCP0.1 + DBDS0.05	0.1	0.05	
TCP0.1 + DBDS0.1	0.1	0.1	
TCP0.1 + DBDS0.025 + OBCS0.025	0.1	0.025	0.025
TCP0.1 + DBDS0.025 + OBCS0.05	0.1	0.025	0.05
TCP0.1 + DBDS0.025 + OBCS0.1	0.1	0.025	0.1
TCP0.1 + DBDS0.05 + OBCS0.025	0.1	0.05	0.025
TCP0.1 + DBDS0.05 + OBCS0.05	0.1	0.05	0.05
TCP0.1 + DBDS0.05 + OBCS0.1	0.1	0.05	0.1
TCP0.1 + DBDS0.1 + OBCS0.025	0.1	0.1	0.025
TCP0.1 + DBDS0.1 + OBCS0.05	0.1	0.1	0.05
TCP0.1 + DBDS0.1 + OBCS0.1	0.1	0.1	0.1
TCP0.05 + DBDS0.025 + OBCS0.05	0.05	0.025	0.05
TCP0.05 + DBDS0.05 + OBCS0.025	0.05	0.05	0.025
TCP0.05 + DBDS0.05 + OBCS0.05	0.05	0.05	0.05
TCP0.05 + DBDS0.05 + OBCS0.1	0.05	0.05	0.1
TCP0.05 + DBDS0.1 + OBCS0.05	0.05	0.1	0.05
TCP0.025 + DBDS0.05 + OBCS0.025	0.025	0.05	0.025
TCP0.025 + DBDS0.05 + OBCS0.05	0.025	0.05	0.05
TCP0.025 + DBDS0.05 + OBCS0.1	0.025	0.05	0.1

### Friction test procedures

Friction was measured using a laboratory-made high-frequency reciprocating tribometer (HFRT)^45^. A precision steel ball with a 3/8-inch diameter and a cylindrical rod with an 8-mm diameter and an 8-mm length were utilized as the ball and disk specimens, respectively. Both specimens were made of heat-treated, high-carbon chromium-bearing steel in accordance with JIS-SUJ2 (AISI51200 steel). The end surface of the as-received cylindrical rod was burnished with a lapping machine (Maruto ML-482A), ending in an isotropically smooth surface with an arithmetic average roughness (Ra) of approximately 0.02 μm. Before the HFRT test, both specimens were ultrasonically cleaned in a toluene bath for 15 min twice, then dried and cleaned with UV-ozone for 25 min.

The ball specimen, fixed to an upper specimen holder, moved with a linear back-and-forth motion at a frequency of 15 Hz and a stroke length of 2.8 mm. When the sample oil reached the test temperature (100 °C), the disk specimen was pressed against the ball specimen with a normal load (29 N), leading to a maximum contact pressure of 1.4 GPa, and then the friction test began. Friction coefficients at the stroke of the ball were acquired as a function of the ball position. Since we considered that the sliding speed was almost constant in the range of ± 1.1 mm from the center of the stroke, the coefficients of friction were averaged in this range. The average coefficient of friction was obtained as a function of the sliding distance. After a 4-h friction test, the disk specimen was cleaned with toluene, and the remaining toluene was blown off with compressed dry air.

### Analysis

The elemental distributions of tribofilms formed from lubricant additives were analyzed by EPMA. The EPMA was conducted on a JEOL JXA-8200 SuperProbe instrument and operated with an acceleration voltage of 15 keV, a beam current of 100 nA, and a beam diameter of 2 μm. Chemical mapping images were obtained for phosphorus (P), sulfur (S), and calcium (Ca). The whole area of a mapping image was 400 μm × 400 μm, congruent with 200 × 200 pixels (40,000 pixels). It encompassed the whole width of the wear track vertical to the sliding direction.

### Output and input data

The friction coefficient at the end of the friction test was used as the output data. The CNN model classified the image data of the elemental distributions of tribofilms into a high friction-coefficient group and a low friction-coefficient group. The friction tests showed that calcium tribofilms formed on the friction surface and that the addition of OBCS increased the friction coefficient. The friction coefficients of most sample oils with OBCS were 0.105 or higher, while those of most sample oils without OBCS were 0.105 or lower. Therefore, the high group and the low group were separated by a friction coefficient of 0.105. On the other hand, the P, S, and Ca mapping images obtained by EPMA were used as the input data. These images were converted to grayscale images according to the maximum value of each elemental intensity, and the image size was changed to fit the CNN model. The grayscale images from the elemental mapping images were mapped to red, green, and blue to generate color images of the RGB color model. In this study, three types of image datasets were prepared: a P–P–P image dataset from three phosphorus mapping images; a P–S–Ca image dataset from a phosphorus, a sulfur, and a calcium mapping image; and a Ca–Ca–Ca image dataset from three calcium mapping images. The image data rotated 180° were also used to increase the total amount of data used for the CNN model. As a result, the number of images generated was 72, twice the number of sample oils.

### Construction of the CNN model

The CNN model in this study was a residual neural network (ResNet), a type of CNN^46^. CNNs are commonly used for image datasets, and ResNets have been used in many studies because they improve the vanishing gradient problem. There are several types of ResNets, depending on the number of hidden layers; ResNet18 was used in this study. The image dataset was randomly divided into two parts at a 4:1 ratio, and these were adopted as the training and test datasets, respectively. The CNN model was trained on the former, and the performance of the CNN model was evaluated based on the accuracy of the latter.

## Results and discussion

### Friction test results

**Figure 2:**
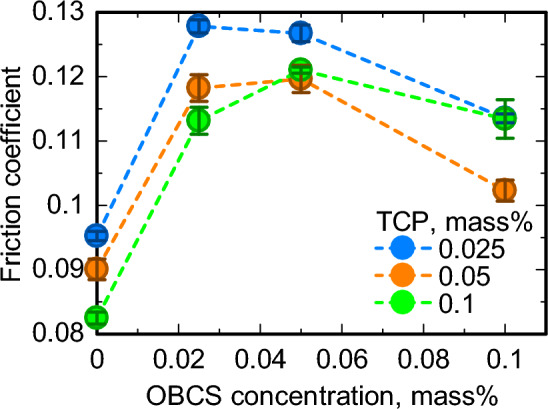
From the two friction test results for the varying concentrations of OBCS at each TCP concentration with a DBDS elemental concentration of 0.05 mass%, the addition of OBCS increased the friction coefficient.

**Figure 3:**
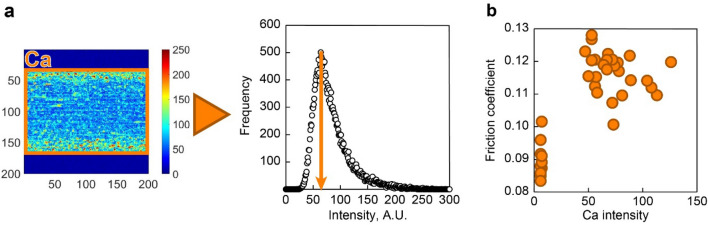
The method for calculating the mode value of Ca intensity from the elemental mapping image obtained through EPMA (**a**) and the results for the relationship between the mode value of Ca intensity and the friction coefficient (**b**). The tribofilms formed from OBCS had high friction coefficients.

First, the friction test of each sample oil was evaluated for the friction coefficient. The friction tests were performed twice for each sample oil to confirm reproducibility. Figure 2 shows the friction coefficient results for the change in OBCS concentration at each TCP concentration at a DBDS elemental concentration of 0.05 mass%. As can be seen in Fig. 2, the addition of OBCS increased the friction coefficient. One of the friction test results for each sample oil was used to construct the machine learning models. Furthermore, the presence of calcium compounds on the friction surface was confirmed from the calcium mapping image of the tribofilms formed from the sample oils by EPMA. A histogram analysis was performed to extract the intensity of each element within the wear track in the mapping image, and then the mode value of Ca intensity was calculated from the histogram^47,48^. Figure 3 shows the method for calculating the mode value of Ca intensity as well as the results for the relationship between the mode value of Ca intensity and the friction coefficient. As the figure shows, the samples in which the mode value of Ca intensity was confirmed had high friction coefficients. Therefore, the friction test results suggested that the calcium tribofilms formed on the friction surface and that the addition of OBCS increased the friction coefficient. The friction coefficients of most sample oils with OBCS were 0.105 or higher, while those of most sample oils without OBCS were 0.105 or lower. We investigated whether the CNN model was able to recognize the tribofilms formed from OBCS and classify the image data of the elemental distributions of tribofilms into the high friction-coefficient group, in which the friction coefficient was higher than 0.105, and the low friction-coefficient group, in which the friction coefficient was lower than 0.105.

### CNN model performance

**Figure 4:**
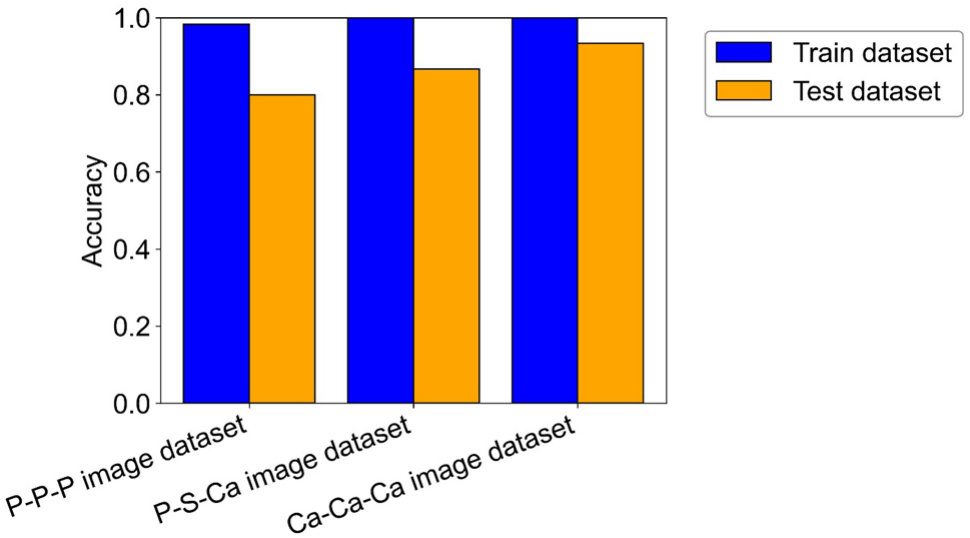
ResNet18 models were constructed to classify image datasets into high and low friction-coefficient groups. High prediction accuracy was achieved for the test datasets of the P–S–Ca and Ca–Ca–Ca image datasets.

**Figure 5:**
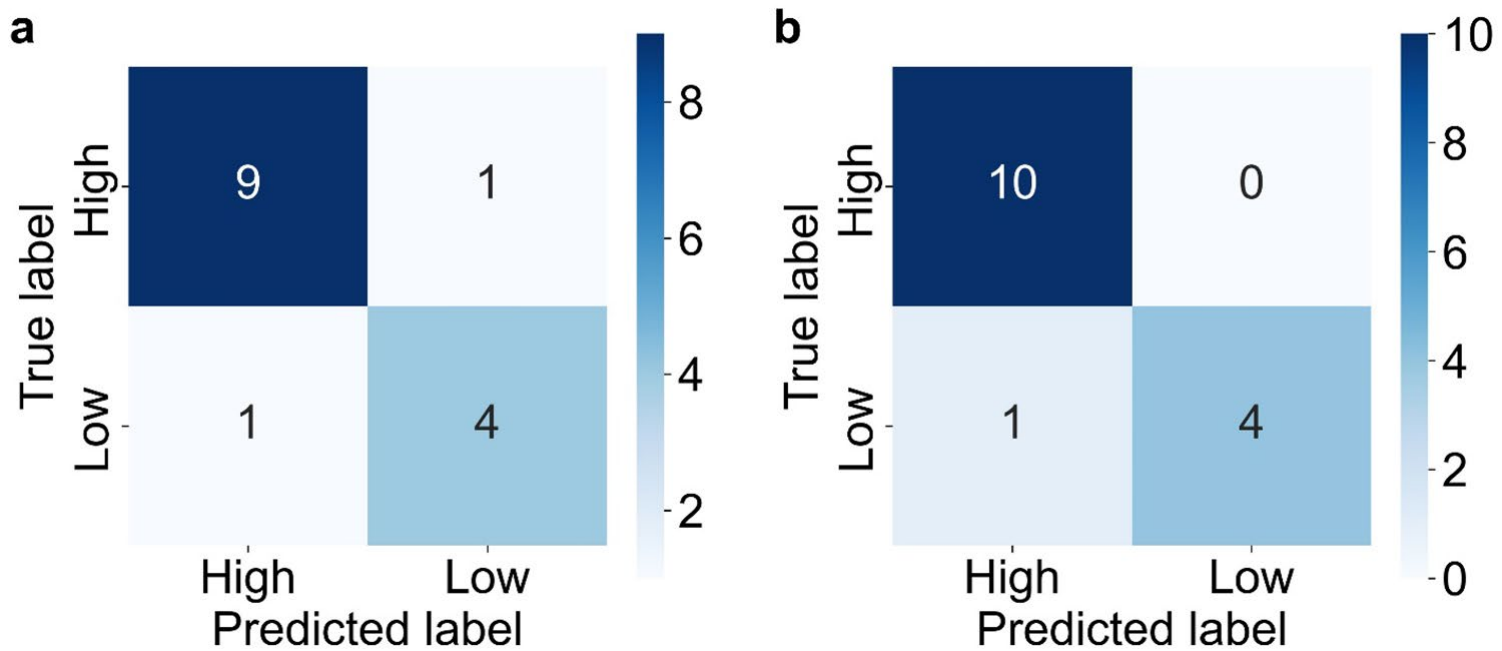
Confusion matrix results for the test datasets of the P–S–Ca image dataset (**a**) and the Ca–Ca–Ca image dataset (**b**). The ResNet18 models successfully separated the test datasets into high and low friction-coefficient groups.

We investigated the CNN model’s performance in classifying datasets into two friction coefficient groups. The hyperparameters of the CNN model were manually adjusted to improve prediction accuracy, and performance was evaluated based on the accuracy for the test dataset of each image dataset. Figure 4 show the accuracy results for the training and test datasets within each image dataset. Table 2 shows the hyperparameters of the ResNet18 model. From Fig. 4, we can see that high prediction accuracy of 0.8 or higher was achieved for all of the image dataset and that the accuracy for the test datasets of the P–S–Ca and Ca–Ca–Ca image datasets was greater than that of the P–P–P image dataset. This suggested that the CNN model recognized the tribofilms formed from OBCS, and was able to classify the test datasets into high and low friction-coefficient groups with high accuracy. Furthermore, Fig. 5 shows the confusion matrix results for the test datasets of the P–S–Ca and Ca–Ca–Ca image datasets. Each row of the confusion matrix represents a true label, while each column of the confusion matrix represents a predicted label. The confusion matrix allows you to see whether the labels predicted by the CNN model match the actual label for each sample data point. From Fig. 5, the CNN model successfully separated the test dataset into high and low friction-coefficient groups.

**Table 2:** The hyperparameters of the ResNet18 model used to classify the datasets into two friction coefficient groups to improve prediction accuracy.

ResNet18	Hyperparameter
Criterion	Cross entropy loss
Optimizer	Adam
Learning rate	0.02
Batch size	4
Epoch	100

### Results of Grad-CAM for the CNN model

**Figure 6:**
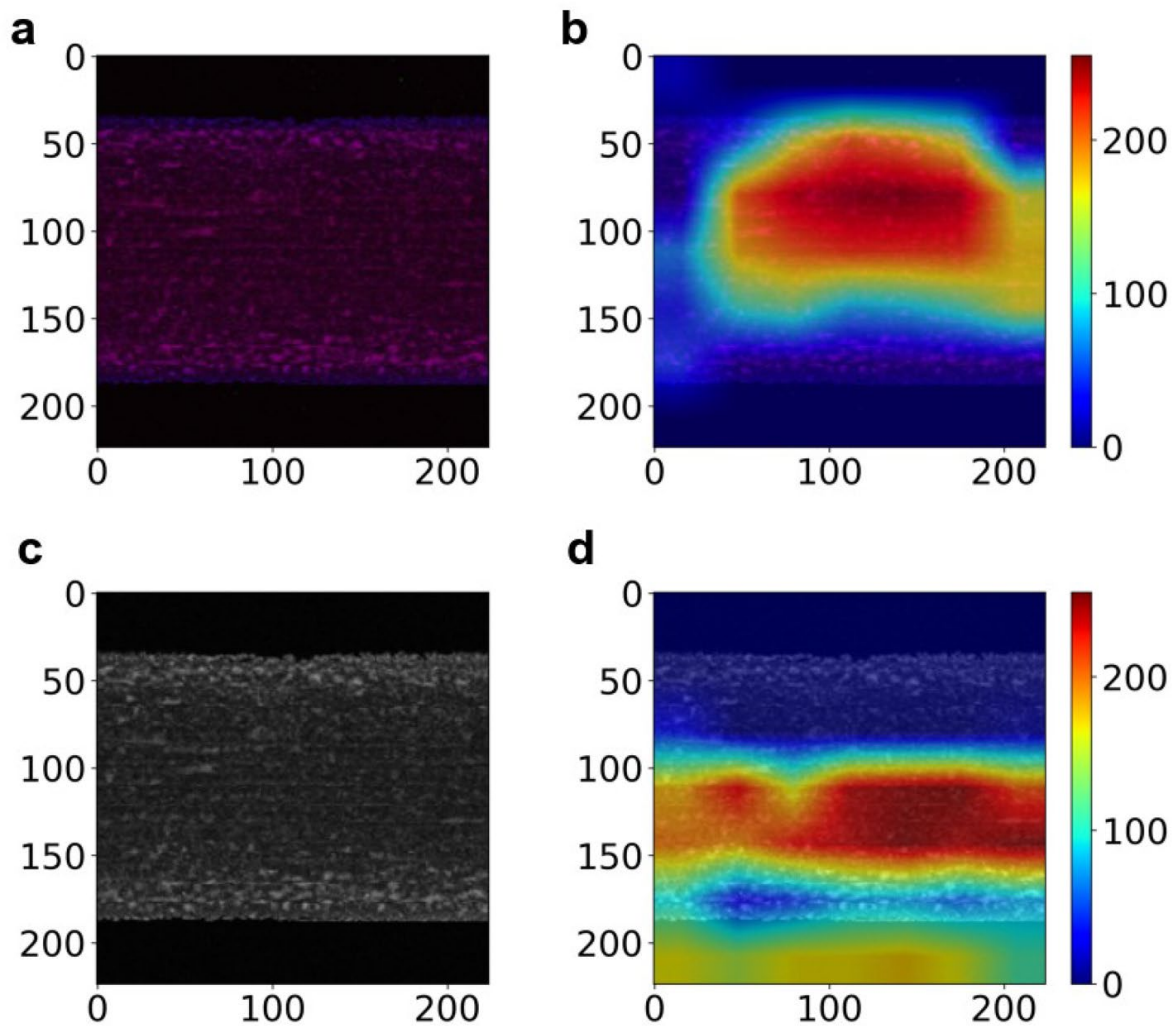
Grad-CAM visualized the features in the sample image that were important for classification into the two friction coefficient groups. The sample image data (**a**) and the Grad-CAM results (**b**) in the high friction-coefficient group of the P–S–Ca image dataset, and the sample image data (**c**) and the Grad-CAM results (**d**) in the high friction-coefficient group of the Ca–Ca–Ca image dataset. The Grad-CAM results indicated that the tribofilms formed from OBCS was important for classifying datasets into the two friction coefficient groups.

Grad-CAM was conducted to visualize which areas are important for classifying datasets into the two friction coefficient groups^40^. Figure 6 shows the sample data along with the Grad-CAM results in the high friction-coefficient groups of the P–S–Ca and Ca–Ca–Ca image datasets. From Fig. 6b,d, it can be seen that the areas on the tribofilms formed from OBCS had high intensity in the Grad-CAM results, underscoring their significance in classifying the image data of the elemental distributions of the tribofilms into high and low friction-coefficient groups. This indicated that the CNN model recognized the tribofilms formed from OBCS and used this information to classify them into two groups based on their friction coefficients.

### Results of dimension reduction of image data

**Figure 7:**
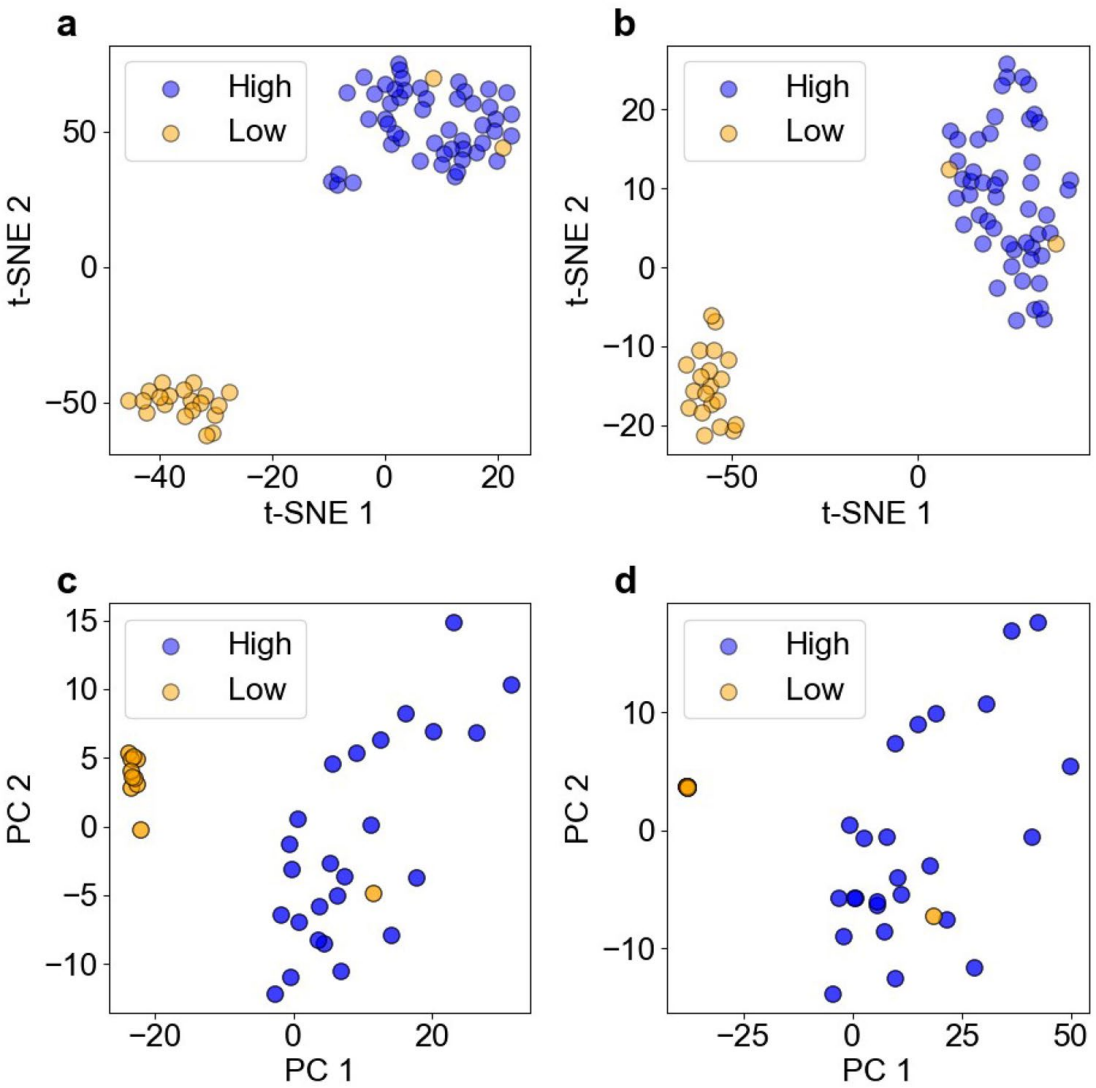
Dimension reductions reduced the number of dimensions of the image data and visualized the distribution of the high-dimensional image dataset. The t-SNE results for the P–S–Ca image dataset (**a**) and the Ca–Ca–Ca image dataset (**b**), and the PCA results for the P–S–Ca image dataset (**c**) and the Ca–Ca–Ca image dataset (**d**). The dimension reductions clearly separated the image datasets into high and low friction-coefficient groups.

Dimensions were reduced to visualize the elemental distributions of high-dimensional image datasets. To reduce the dimensions, we used t-distributed stochastic neighbor embedding (t-SNE) and principal component analysis (PCA)^49^. t-SNE and PCA can visualize the distribution of a high-dimensional image dataset by creating new axes. Figure 7a,b show the t-SNE results of the P–S–Ca and Ca–Ca–Ca image datasets. The horizontal and vertical lines represent the first and second features of the t-SNE. The parameters of the t-SNE results are shown below: the perplexity (5), the early exaggeration (12), the learning rate (50), and the iterations (20,000). From Fig. 3, it was confirmed that both the P–S–Ca and Ca–Ca–Ca image datasets were clearly separated into high and low groups. These results indicated that the t-SNE classification into two friction coefficient groups was successful. Figure 7c,d show the PCA results of the P–S–Ca and Ca–Ca–Ca image datasets. The horizontal and vertical lines represent the first and second principal components of the PCA, respectively. As can be seen, the samples in the low group were located close to each other, and then image data overlapped with the image data that had been rotated 180°, while the samples in the high group were widely distributed vertically. This suggested that the first principal component separated the image datasets into high and low friction-coefficient groups depending on the Ca intensity of the tribofilms, while the second principal component placed the samples in the high group in various locations as it recognized other features of the tribofilms. Therefore, the PCA results suggested that while PCA could classify the datasets into high and low friction coefficient groups, it was less distinct than t-SNE in its separation of the samples into high and low groups.

## Conclusions

In this study, a CNN model was constructed to investigate the relationship between the elemental distribution of a tribofilm formed from phosphorus-, sulfur-, and calcium-based additives on the one hand and the coefficient of friction on the other. The CNN model was designed to classify the distributions into high and low friction coefficient groups based on mapping images of P, S, and Ca obtained through EPMA. We were able to draw the following conclusions from the results of the friction coefficient classification and the analyses of the CNN model.

1. The friction tests demonstrated the creation of calcium tribofilms on the friction surface, with the addition of OBCS markedly increasing the friction coefficient. Therefore, the CNN model was constructed to classify the image data of the elemental distributions of tribofilms into high and low friction-coefficient groups.
2. The results of the friction coefficient classification demonstrated that the ResNet18 model could classify elemental distributions into high and low friction coefficient groups with high prediction accuracy. Furthermore, the accuracy off the test datasets for the P–S–Ca and Ca–Ca–Ca image datasets was higher than that of the P–P–P image dataset. This suggested that the CNN model recognized the tribofilms formed from OBCS and was able to classify the test datasets into high and low friction-coefficient groups with high accuracy.
3. Grad-CAM was able to visualize which areas are important for classification into the two friction coefficient groups. The results of the Grad-CAM of the P–S–Ca and Ca–Ca–Ca image datasets suggested that the areas on the tribofilms formed from OBCS were important for classification into the two friction coefficient groups. This indicated that the CNN model recognized the tribofilms formed from OBCS to classify the datasets into the two friction coefficient groups.
4. The dimension reductions enabled the visualization of image data distribution. The results of the dimension reductions of the P–S–Ca and Ca–Ca–Ca image datasets suggested that both were divided into high and low friction-coefficient groups. Furthermore, the results showed that, compared to PCA, t-SNE more clearly divided the data into high and low groups.

The results of this study indicate that the CNN model, the Grad-CAM, and the dimension reductions are useful for evaluating the frictional features of tribofilms formed from multiple lubricant additives.

## Data Availability

The datasets generated and analyzed in the current study are not publicly available because the datasets will be used in our future research. However, they are available from the corresponding author on reasonable request.

## References

[1] Holmberg K, Andersson P, Erdemir A (2012). Global energy consumption due to friction in passenger cars. Tribol. Int..

[2] Wong VW, Tung SC (2016). Overview of automotive engine friction and reduction trends—Effects of surface, material, and lubricant-additive technologies. Friction..

[3] Narita K (2012). Tribological properties of metal V-belt type CVT lubricant. Adv. Tribol..

[4] Narita K (2014). Lubricants for metal belt continuously variable transmissions. Lubricants..

[5] Bieber HE, Klaus EE, Tewksbury EJ (1968). A study of tricresyl phosphate as an additive for boundary lubrication. ASLE. Trans..

[6] Godfrey D (1965). The lubrication mechanism of tricresyl phosphate on steel. ASLE. Trans..

[7] Spikes HA, Cameron A, Gisser H, Goldblat IL (1974). Additive interference in dibenzyl disulfide extreme pressure lubrication. ASLE. Trans..

[8] Plaza S (1989). The studies of dibenzyl disulfide tribochemical reactions in the presence of other additives. Tribol. Trans..

[9] Bovington CH, Dacre B (1982). Thermal decomposition of dibenzyl disulfide in hexadecane. ASLE. Trans..

[10] Topolovec-Miklozic K, Forbus TR, Spikes H (2008). Film forming and friction properties of overbased calcium sulphonate detergents. Tribol. Lett..

[11] Zhu L, Zhao G, Wang X (2017). Investigation on three oil-miscible ionic liquids as antiwear additives for polyol esters at elevated temperature. Tribol. Int..

[12] Massoud T (2020). Effect of ZDDP on lubrication mechanisms of linear fatty amines under boundary lubrication conditions. Tribol. Int..

[13] De Barros-Bouchet MI (2015). Tribochemistry of phosphorus additives: Experiments and first-principles calculations. RSC. Adv..

[14] Spadaro F, Rossi A, Laine E, Woodward P, Spencer ND (2017). Elucidating the resistance to failure under tribological tests of various boron-based films by XPS and ToF-SIMS. Appl. Surf. Sci..

[15] Wang C, Gojzewski H, Schipper DJ (2020). A multi-technique characterization of the tribofilm formed by a fully formulated CVT fluid. Tribol. Int..

[16] Pandiyan V (2023). Long short-term memory based semi-supervised encoder-decoder for early prediction of failures in self-lubricating bearings. Friction..

[17] Prost J (2021). Semi-supervised classification of the state of operation in self-lubricating journal bearings using a random forest classifier. Lubricants..

[18] Song Q, Zhao S, Wang M (2020). On the accuracy of fault diagnosis for rolling element bearings using improved DFA and multi-sensor data fusion method. Sensors..

[19] Wang X, Mao D, Li X (2021). Bearing fault diagnosis based on vibro-acoustic data fusion and 1D-CNN network. Measurement..

[20] Prost J, Boidi G, Puhwein AM, Varga M, Vorlahufer G (2023). Classification of operational states in porous journal bearings using a semi-supervised multi-sensor machine learning approach. Tribol. Int..

[21] Stebakov I, Kornaev A, Popov S, Savin L (2022). Fault diagnosis systems for rotating machines operating with fluid-film bearings. Proc. Inst. Mech. Eng. Part. J. J. Eng. Tribol..

[22] Desai PS, Granja V, Higgs CF (2021). Lifetime prediction using a tribology-aware, deep learning-based digital twin of ball bearing-like tribosystems in oil and gas. Processes..

[23] Wescoat E, Bradford J, Krugh M, Mears L (2022). Contamination factor prediction using contrived data for bearing useful life estimation. Manuf. Lett..

[24] Moder J, Bergmann P, Grun F (2018). Lubrication regime classification of hydrodynamic journal bearings by machine learning using torque data. Lubricants..

[25] Mokhtari N, Pelham JG, Nowoisky S, Bote-Garcia JL, Guhmann C (2020). Friction and wear monitoring methods for journal bearings of geared turbofans based on acoustic emission signals and machine learning. Lubricants..

[26] Hasan MS, Kordijazi A, Rohatgi PK, Nosonovsky M (2022). Machine learning models of the transition from solid to liquid lubricated friction and wear in aluminum-graphite composites. Tribol. Int..

[27] Ulas M, Altay O, Gurgenc T, Ozel C (2020). A new approach for prediction of the wear loss of PTA surface coatings using artificial neural network and basic, kernel-based, and weighted extreme learning machine. Friction..

[28] Altay O, Gurgenc T, Ulas M, Ozel C (2020). Prediction of wear loss quantities of ferro-alloy coating using different machine learning algorithms. Friction..

[29] Bustillo A, Pimenov DY, Matuszewski M, Mikolajczyk T (2018). Using artificial intelligence models for the prediction of surface wear based on surface isotropy levels. Robot. Comput. Integr. Manuf..

[30] Gangwar S, Pathak VK (2020). Dry sliding wear characteristics evaluation and prediction of vacuum casted marble dust (MD) reinforced ZA-27 alloy composites using hybrid improved bat algorithm and ANN. Mater. Today. Commun..

[31] Hasan MS, Kordijazi A, Rohatgi PK, Nosonovsky M (2021). Triboinformatic modeling of dry friction and wear of aluminum base alloys using machine learning algorithms. Tribol. Int..

[32] Bustillo A, Reis R, Machado AR, Pimenov DY (2022). Improving the accuracy of machine-learning models with data from machine test repetitions. J. Intell. Manuf..

[33] Kumar R, Chauhan S (2015). Study on surface roughness measurement for turning of Al 7075/10/SiCp and Al 7075 hybrid composites by using response surface methodology (RSM) and artificial neural networking (ANN). Measurement..

[34] Wang H, Zhang C, Yu X, Li Y (2023). Evaluating wear volume of oligoether esters with an interpretable machine learning approach. Tribol. Lett..

[35] Dai K, Gao X (2013). Estimating antiwear properties of lubricant additives using a quantitative structure tribo-ability relationship model with back propagation neural network. Wear..

[36] Rosenkranz A, Marian M, Profito FJ, Aragon N, Shah R (2021). The use of artificial intelligence in tribology—A perspective. Lubricants..

[37] Shah R (2023). Ensemble deep learning for wear particle image analysis. Lubricants..

[38] Sharma K, Goyal D, Kanda R (2022). Intelligent fault diagnosis of bearings based on convolutional neural network using infrared thermography. Proc. Inst. Mech. Eng. Part. J. J. Eng. Tribol..

[39] Liu Z (2023). Intelligent classification of online wear particle in lubricating oil using optical direct imaging method and convolutional neural network for rotating machinery. Tribol. Int..

[40] Selvaraju RR (2020). Grad-CAM: Visual explanations from deep networks via gradient-based localization. Int. J. Comput. Vis..

[41] Park S, Wallraven C (2022). Comparing facial expression recognition in humans and machines: Using CAM, GradCAM, and extremal perturbation. Pattern. Recogn..

[42] Ghadai S, Balu A, Sarkar S, Krishnamurthy A (2018). Learning localized features in 3D CAD models for manufacturability analysis of drilled holes. Comput. Aided. Geom. Des..

[43] Stachowiak GP, Podsiadlo P, Stachowiak GW (2006). Evaluation of methods for reduction of surface texture features. Tribol. Lett..

[44] Bolelli G (2015). Tribology of HVOF- and HVAF-sprayed WC-10Co4Cr hardmetal coatings: A comparative assessment. Surf. Coat. Technol..

[45] Itoga M (2016). Toward resolving anxiety about the accelerated corrosive wear of steel lubricated with the fluorine-containing ionic liquids at elevated temperature. Tribol. Int..

[46] He, K., Zhang, X., Ren, S. & Sun, J. Deep residual learning for image recognition. 10.48550/arXiv.1512.03385.

[47] Matsui Y, Aoki S, Masuko M (2016). Influence of coexisting functionalized polyalkylmethacrylates on the formation of ZnDTP-derived tribofilm. Tribol. Int..

[48] Sato T, Aoki S, Masuko M (2017). Determination of the inherent friction characteristic of ZnDTP-derived tribofilms formed inhomogeneously over the contact surfaces. Tribol. Int..

[49] van der Maaten L, Hinton G (2008). Visualizing data using t-SNE. J. Mach. Learn. Res..

